# Biallelic variants in *OGDH* encoding oxoglutarate dehydrogenase lead to a neurodevelopmental disorder characterized by global developmental delay, movement disorder, and metabolic abnormalities

**DOI:** 10.1016/j.gim.2022.11.001

**Published:** 2023-02

**Authors:** Ella F. Whittle, Madison Chilian, Ehsan Ghayoor Karimiani, Helga Progri, Daniela Buhas, Melis Kose, Rebecca D. Ganetzky, Mehran Beiraghi Toosi, Paria Najarzadeh Torbati, Reza Shervin Badv, Ivan Shelihan, Hui Yang, Houda Zghal Elloumi, Sukyeong Lee, Yalda Jamshidi, Alan M. Pittman, Henry Houlden, Erika Ignatius, Shamima Rahman, Reza Maroofian, Wan Hee Yoon, Christopher J. Carroll

**Affiliations:** 1 Genetics Section, Molecular and Clinical Sciences Research Institute, St. George’s, University of London, London, United Kingdom; 2 Aging & Metabolism Research Program, Oklahoma Medical Research Foundation, Oklahoma City, OK; 3 Innovative Medical Research Centre, Faculty of Medicine, Islamic Azad University-Mashhad Branch, Mashhad, Iran; 4 Division of Medical Genetics, Department of Specialized Medicine, McGill University Health Center, Department of Human Genetics, McGill University, Montreal, Canada; 5 Division of Inborn Errors of Metabolism, Department of Pediatrics, Izmir Katip Çelebi University Faculty of Medicine, Izmir, Turkey; 6 Mitochondrial Medicine Frontier Program, Division of Human Genetics, Children’s Hospital of Philadelphia, Philadelphia, PA; 7 Department of Pediatrics, Perelman School of Medicine, University of Pennsylvania, Philadelphia, PA; 8 Department of Pediatrics, School of Medicine, Mashhad University of Medical Sciences, Mashhad, Iran; 9 Neuroscience Research Center, Mashhad University of Medical Sciences, Mashhad, Iran; 10 Department of Medical Genetics, Next Generation Genetic Polyclinic, Mashhad Iran; 11 Children’s Medical Center, Pediatrics Center of Excellence, Tehran University of Medical Sciences, Tehran, Iran; 12 Division of Medical Genetics, Department of Specialized Medicine, McGill University Health Centre, Montreal, Canada; 13 GeneDx, Gaithersburg, MD; 14 Verna and Marrs McLean Department of Biochemistry and Molecular Biology, Baylor College of Medicine, Houston, TX; 15 Department of Neuromuscular Disorders, UCL Institute of Neurology, University College London, London, United Kingdom; 16 Division of Child Neurology, Children’s Hospital, University of Helsinki and Helsinki University Hospital, Helsinki, Finland; 17 Mitochondrial Research Group, UCL Great Ormond Street Institute of Child Health, and Metabolic Unit, Great Ormond Street Hospital for Children NHS Foundation Trust, London, United Kingdom

**Keywords:** α-ketoglutarate dehydrogenase deficiency, Mitochondria, Neurodevelopmental disease, OGDH, Oxoglutarate dehydrogenase

## Abstract

**Purpose:**

This study aimed to establish the genetic cause of a novel autosomal recessive neurodevelopmental disorder characterized by global developmental delay, movement disorder, and metabolic abnormalities.

**Methods:**

We performed a detailed clinical characterization of 4 unrelated individuals from consanguineous families with a neurodevelopmental disorder. We used exome sequencing or targeted-exome sequencing, cosegregation, in silico protein modeling, and functional analyses of variants in HEK293 cells and *Drosophila melanogaster*, as well as in proband-derived fibroblast cells.

**Results:**

In the 4 individuals, we identified 3 novel homozygous variants in oxoglutarate dehydrogenase (*OGDH*) (NM_002541.3), which encodes a subunit of the tricarboxylic acid cycle enzyme α-ketoglutarate dehydrogenase. In silico homology modeling predicts that c.566C>T:p.(Pro189Leu) and c.890C>A:p.(Ser297Tyr) variants interfere with the structure and function of OGDH. Fibroblasts from individual 1 showed that the p.(Ser297Tyr) variant led to a higher degradation rate of the OGDH protein. OGDH protein with p.(Pro189Leu) or p.(Ser297Tyr) variants in HEK293 cells showed significantly lower levels than the wild-type protein. Furthermore, we showed that expression of *Drosophila Ogdh* (*dOgdh*) carrying variants homologous to p.(Pro189Leu) or p.(Ser297Tyr), failed to rescue developmental lethality caused by loss of *dOgdh*. SpliceAI, a variant splice predictor, predicted that the c.935G>A:p.(Arg312Lys)/p.(Phe264_Arg312del) variant impacts splicing, which was confirmed through a mini-gene assay in HEK293 cells.

**Conclusion:**

We established that biallelic variants in *OGDH* cause a neurodevelopmental disorder with metabolic and movement abnormalities.

## Graphical abstract

**Figure:**
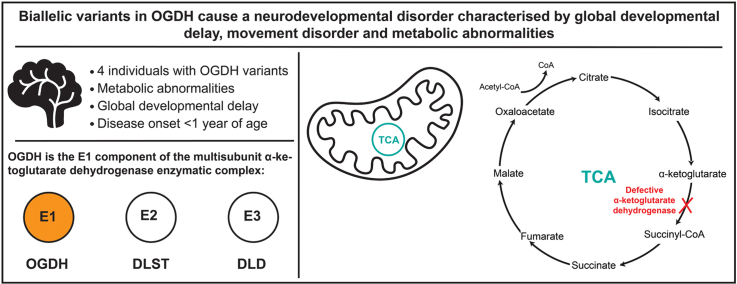

## Introduction

**Figure 1:**
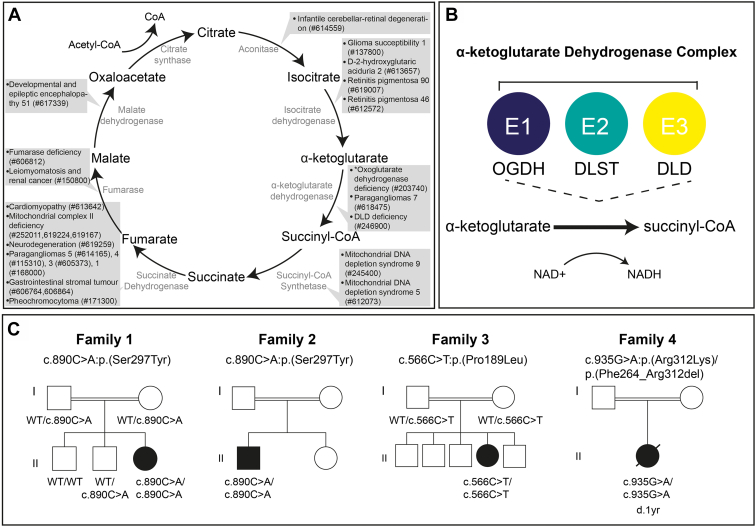
**The TCA cycle, α-ketoglutarate complex enzymatic reaction and family pedigrees of investigated families.** A. The TCA cycle with the OMIM phenotypes associated to each enzymatic complex listed. ∗ indicates OGDH deficiency that is described in this study. B. A schematic of the TCA cycle enzymatic reaction that OGDH catalyzes alongside DLD and DLST as the α-ketoglutarate dehydrogenase complex. C. Pedigrees of the 4 families harboring pathogenic *OGDH* variants. Affected individuals are indicated via filled symbols with implicated variant stated below. DLD, dihydrolipoamide dehydrogenase; DLST, dihydrolipoamide S-succinyltransferase; NAD, nicotinamide adenine dinucleotide; NADH, nicotinamide adenine dinucleotide hydrogen; OGDH, Oxoglutarate dehydrogenase; TCA, tricarboxylic acid.

Aberrant tricarboxylic acid (TCA) cycle enzymes lead to severe pediatric-onset neurologic disorders characterized by metabolic abnormalities (Figure 1A).^1^, ^2^, ^3^, ^4^, ^5^, ^6^, ^7^, ^8^

Oxoglutarate dehydrogenase (OGDH) (OMIM 613022) is an E1 component of the α-ketoglutarate dehydrogenase (α-KGDH) complex, a multisubunit enzyme consisting of OGDH, dihydrolipoamide S-succinyltransferase (DLST) (OMIM 126063), and dihydrolipoamide dehydrogenase (DLD) (OMIM 238331). α-KGDH catalyzes the conversion of α-ketoglutarate to succinyl-coenzyme A (succinyl-CoA) in the TCA cycle (Figure 1B). OGDH requires the cofactor thiamine pyrophosphate (TPP) to decarboxylate α-ketoglutarate and transfer the intermediate substrate to DLST, the E2 component of the complex, during the conversion of α-ketoglutarate to succinyl-CoA.^9^ α-KGDH deficiency (ORPHA:31; OMIM 203740) leads to a recessive neurometabolic disorder defined by cognitive impairment (HP:0100543), movement disorder (HP:0100022), lactic acidosis (HP:0003128), and increased urine α-ketoglutarate concentration (HP:0012402).^10^^,^^11^ Until recently, only pathogenic variants in the α-KGDH member *DLD* had been associated with α-KGDH deficiency. Most pathogenic *DLD* variants cause DLD deficiency (OMIM 246900), an autosomal recessive metabolic disease, of which α-KGDH deficiency presents as part of the disease phenotype.^12^^,^^13^ Two siblings with a homozygous *OGDH* variant have recently been reported with neurologic features consistent with those documented in α-ketoglutarate deficiency.^4^ However, additional cases are required to firmly establish the causality of the *OGDH* variants in human disease.

In this article, we present 4 unrelated families with 3 novel pathogenic *OGDH* variants. Individuals within our study present with clinical features consistent with α-ketoglutarate deficiency and those described in the already reported family with OGDH deficiency.^4^ Experimental data derived from homology modeling, proband-derived fibroblasts, overexpression of OGDH variants in HEK293 cells, exon trapping, and a *Drosophila* disease model further supported the pathogenicity of *OGDH* variants in neurodevelopmental disease.

## Materials and Methods

### Recruitment and data collection

This study included 4 individuals from 4 unrelated families (Table 1). Individuals were recruited after clinical assessment at respective centers, and the cohort was established via GeneMatcher.^14^ Informed parental consent was given for the genetic investigation into the individuals and publication of subsequent findings as per each center’s standard protocol. Ethical approval was obtained for the study and is expanded upon in the Ethics Declaration section. Human samples, blood samples from all individuals and fibroblast samples from individual 1 only, were obtained with written informed consent. Genomic DNA was extracted from proband blood samples at each center and exome sequencing or targeted exome sequencing was performed in accordance with respective laboratory guidelines and protocols. For individual 3, trio sequencing was performed using AIDX and MitoX gene panels according to GeneDx protocols, following clinical suspicion of a mitochondrial disorder.

**Table 1:** Clinical findings in individuals with homozygous OGDH pathogenic variants, including already reported homozygous variant p.Asn320Ser^4^

General and Clinical Information	Individual 1	Individual 2	Individual 3	Individual 4	Individual 5 Yap et al^4^	Individual 6 Yap et al^4^
Sex	F	M	F	F	M	F
Variant	c.890C>A,p.(Ser297Tyr)	c.890C>A p.(Ser297Tyr)	c.566C>T p.(Pro189Leu)	c.935G>A p.(Arg312Lys)/p.(Phe264_Arg312del)^a^	c.959A>G p.(Asn320Ser)	c.959A>G p.(Asn320Ser)
Homozygous	+	+	+	+	+	+
Variant transcript	NM_002541	NM_002541	NM_002541	NM_002541	NM_002541.3	NM_002541.3
Age at presentation	4 mo	9 mo	6 wk	2 mo	3 y	8 mo
Age at last evaluation	4 y	3 y 5 months	3 y 2 mo	Deceased at 12 mo	6 y	17 y
Global developmental delay HP:0001263	+	+	+	+	+	+
Seizures HP:0001250	+	+	–	–	–	+
Hypotonia HP:0001252	+	+	+	+	+	+
Dystonia HP:0001332	+	+	+	–	+	+
Ataxia HP:0001251	+	+	–	–	+	–
Abnormal nasal bridge morphology HP:0000422	–	+	+	+	–	–
Microcephaly HP:0000252	–	+	+	+	NA	NA
Ventriculomegaly HP:0002119	+	+	–	–	+	–
Brain atrophy HP:0012444	+	+	–	–	–	+
Basal ganglia abnormality HP:0002134	–	–	+	–	–	+
Corpus callosum agenesis HP:0001274	+	–	+	–	–	–
Metabolic acidosis HP:0001942	+	+	+	+	NA	NA
Hyperglutaminemia HP:0003217	+	NA	+	+	NA	–
Hyperammonemia HP:0001987	+	+	–	+	–	–
Increased serum lactate HP:0002151	+	+	+	+	+	+
Elevated urinary α-ketoglutarate HP:0012401	NA	NA	+	+	–	–

**Figure 2:**
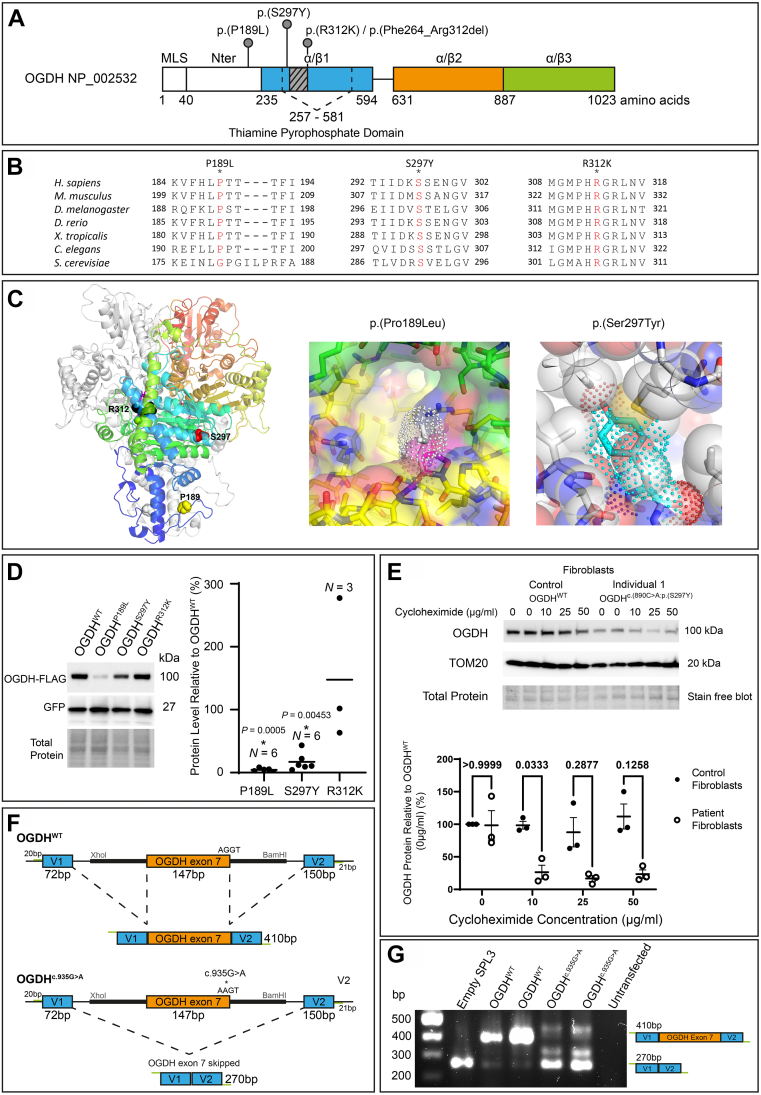
**OGDH protein instability or aberrant splicing demonstrated both in silico and in vitro.** A. A schematic of the OGDH protein (NP_002532.2) with the TPP domain indicated alongside the OGDH variants identified in this study. The α/β1 domain is indicated in blue, α/β2 domain is indicated in orange, and α/β3 domain is indicated in green. We found that the c.935G>A p.[R312K]/p.[Phe264_Arg312del]) variant to impact splicing in our mini-gene assay (F-G), and the resulting deletion is indicated as a gray box in this schematic. In addition, the domain linker is indicated as a line between α/β1 and α/β2. The position of domains are indicated as previously reported.^30^ B. The OGDH variants reported alter evolutionarily conserved amino acids, as can be seen for all 3 variants. C. Homology model of the human OGDH dimer showing the location of the missense variants. Left: OGDH dimer is depicted as ribbon diagram with one monomer shown in white and the other in rainbow color from red (N-terminus) to blue (C-terminus). The bound TPP cofactor is shown as stick model (magenta) and variant sites as Corey-Pauling-Koltun model. Middle: Pro189 is located at the dimer interface. Residues surrounding Pro189Leu variant shown in yellow (*cis* subunit) and green (*trans* subunit). The bulky hydrophobic side chain of leucine substitution (white stick with dotted surface) likely destabilizes the dimer. Right: Ser297Tyr variant would cause structural clash with surrounding residues. In contrast to the serine side chain of OGDH (sphere), the tyrosine side chain of the OGDH mutant (dotted surface) would clash with neighboring atoms. D. OGDH-FLAG+GFP protein levels, detected using immunoprecipitation, in HEK293 cells transfected with either pcDNA3.1-OGDH^WT^-FLAG, pcDNA3.1-OGDH^p.(Pro189Leu)^-FLAG, pcDNA3.1-OGDH^p.(Ser297Tyr)^-FLAG, or pcDNA3.1-OGDH^p.(Arg312Lys)^-FLAG. GFP and total protein detection used for normalization. Results show a significant reduction in protein as a result of the p.(Pro189Leu) and p.(Ser297Tyr) variants. Kruskal-Wallis and a one-sample Wilcoxon test was used to statistically analyze. E. Endogenous OGDH levels detected using immunoblotting in individual 1 (p.[Ser297Tyr]) fibroblast cells and control fibroblast cells treated with cycloheximide for 24 hours at varying concentrations (0-50 μg/ml). TOM20 protein was detected and used to normalize for total mitochondrial mass within cells. OGDH level was normalized to TOM20 and analyzed as percentage change compared with control OGDH levels produced by control fibroblasts at 0 μg/mL treatment. Cycloheximide treatment revealed a higher rate of protein turnover of the OGDH protein produced by individual 1 fibroblasts than by control fibroblasts. This was significant at 10 μg/mL. *P* values indicated above data points. *N* = 3, two-way ANOVA. F, G. Results of the pSPL3 mini-gene splicing assay showing the c.935G>A variant causes whole exon skipping. F. Schematic showing the design of the pSPL3 vector. *OGDH* exon 7 with flanking intronic sequence indicated by the thick black line was introduced into the vector using Xhol and BamHI restriction enzyme sites. Lines indicate intronic sequence and boxes indicate exons, including the vector exons V1 and V2. *OGDH* exon 7 with wild-type sequence, seen above, results in accurate splicing and removal of introns. An alternative splicing pattern is shown below due to the c.935G>A variant. It results in inaccurate splicing and removal of introns as well as exon 7. Expected reverse transcription polymerase chain reaction (RT-PCR) product sizes are indicated and inclusive of primer sequences. G. HEK293 cells were transfected with the pSPL3-OGDH^WT^, pSPL3-OGDH^c.935G>A^, and empty pSPL3 vector. RNA extracts were obtained and converted to complementary DNA through RT-PCR. PCR products were amplified and visualized on a 1.5% gel. pSPL3-OGDH^WT^ resulted in the correctly spliced product of 410 bp. pSPL3-OGDH^c.935G>A^ did not result in the correctly spliced 410 bp but did result in a 270 bp product, showing that the c.935G>A variant results in whole exon skipping. ANOVA, analyis of variance; bp, basepair; OGDH, oxoglutarate dehydrogenase; TPP, thiamine pyrophosphate.

*F*, female; *M*, male; *NA*, not available; +, feature present; –, feature absent.

a The c.935G>A:p.(Arg312Lys)/p.(Phe264_Arg312del variant was found to affect splicing and cause a deletion in a mini-gene splicing assay (Figure 2F and G).

### Bioinformatics tools

We queried our variants of interest in the population databases Genome Aggregation Database and Iranome.^15^^,^^16^ To assist in variant interpretation, the following in silico pathogenicity predictors were used: Combined Annotation Dependent Depletion (CADD), Sorting Intolerant from Tolerant, Polymorphism Phenotyping (PolyPhen), SpliceAI, and Rare Exome Variant Ensemble Learner (REVEL).^17^, ^18^, ^19^, ^20^, ^21^

### Homology modeling

To generate a homology model for the human OGDH dimer, the atomic coordinates for the SucA domain of *Mycobacterium smegmatis* α-ketoglutarate decarboxylase (Protein Data Bank Identifier: 2Y0P) were used as templates in Modeller.^22^ Superposition of our model and the monomer model predicted by the AlphaFold^23^ gave a root mean square deviation of 0.743 Å over 641 Cα atoms. OGDH variants were generated in silico using COOT^24^ followed by geometry optimization.

### Cell culture and transfection

Skin fibroblasts and HEK293 cells were cultured in Dulbecco’s Modified Eagle’s Medium (D6546, Sigma Aldrich) supplemented with L-Glutamine (G7513, Sigma Aldrich), 10% fetal bovine serum (F9665, Sigma Aldrich), and penicillin streptomycin (P0781, Sigma Aldrich) at 37 °C in a 5% carbon dioxide (CO_2_) atmosphere.

Transfection of HEK293 cells with pcDNA3.1+/C-(K)-DYK-OGDH^WT^, pcDNA3.1+/C-(K)-DYK-OGDH^p.(Pro189Leu)^, pcDNA3.1+/C-(K)-DYK-OGDH^p.(Ser297Tyr)^, or pcDNA3.1+/C-(K)-DYK-OGDH^p.(Arg312Lys)^ together with pcDNA3.1(+)eGFP was performed using a 1:4 polyethylenimine (11460630, Fisher Scientific) to DNA transfection. Protein was extracted using RIPA lysis buffer (24948, Santa Cruz Biotechnology) with protease inhibitors and sodium orthovanadate according to manufacturer’s instructions (24948, Santa Cruz Biotechnology). Samples were incubated on ice for 20 minutes then centrifuged for 20 minutes at 13,000*g* at 4 °C. The supernatant was collected and Laemmli sample buffer (161-0747, Bio-Rad) was added with 2-mercaptoathanol (M3148, Sigma Aldrich) at a final concentration of 5%.

Endogenous OGDH protein was measured from fibroblast cells derived from individual 1 and control fibroblast cells. Both cell lines were plated onto 6 well tissue culture dishes and cultured as described earlier. Cycloheximide (sc-3508, Insight Biotechnology) was added to plate wells at varying final concentrations (0 μg/mL, 10 μg/mL, 25 μg/mL, and 50 μg/mL) and incubated for 24 hours. After incubation, protein was extracted using RIPA lysis buffer as described earlier, and OGDH protein amounts were measured through immunoblotting analysis.

### Immunoblotting analysis

All protein electrophoresis were performed on a 4% to 15% gradient TGX stain free gel (4568083, Bio-Rad) and ran in a Mini-PROTEAN Tetra System (Bio-Rad). Upon completion, the proteins were transferred using a Trans-Blot Turbo Mini size PVDF Transfer Kit (1704272, Bio-Rad) and transferred in a Trans-Blot Turbo Transfer System (Bio-Rad). After transfer, the membrane was blocked in a 5% milk Tris-buffered saline with Tween 20 (TBST) solution at room temperature for an hour and then washed with TBST (0.1% Tween 20) before incubating in a primary antibody solution overnight at 4 °C in 1% bovine serum albumin in TBST. Primary antibodies used were rabbit anti-GFP (1:1000, 50430-2-AP, ProteinTech), rabbit anti-Flag (1:2000, 2368S, Cell Signaling Technology), TOM20 (1:2000, 11802-1-AP, ProteinTech), and OGDH (1:1000, 15212-1-AP, ProteinTech). After overnight incubation, the membrane was incubated with an HRP-conjugated secondary antibody (mouse antirabbit SC2357, 1:10,000, Santa Cruz Biotechnology). Membranes were covered with Clarity enhanced chemiluminescence substrate (1705061, Bio-Rad) and imaged using a Chemidoc MP imaging system (Bio-Rad). Quantification of bands was performed using ImageLab software and statistically analyzed using GraphPad Prism software.

A Kruskal-Wallis test was employed to compare OGDH^p.(Pro189Leu)^-FLAG and OGDH^p.(Ser297Tyr)^-FLAG protein vs OGDH^WT^-FLAG protein isolated from the HEK293 transfection experiment, minus an outlier identified within the data set for p.(Pro189Leu) protein expression. In addition, a one-sample Wilcoxon test was used to compare OGDH^p.(Arg312Lys)^-FLAG protein vs OGDH^WT^-FLAG. To analyze OGDH protein levels, normalized to TOM20, after cycloheximide treatment of individual 1 fibroblasts vs control fibroblasts, values were further normalized to the control OGDH protein isolated from control fibroblast cells and analyzed together using a two-way analysis of variance.

### Exon-trapping mini-gene assay

The pSPL3 splicing vector^25^ was used in an exon-trapping assay to confirm the aberrant splicing caused by the suspected pathogenic OGDH c.(935G>A):p.(Arg312Lys) variant. *OGDH* exon 7 containing either the c.(935G>A) variant or the reference allele was introduced into the pSPL3 plasmid via the Xhol and BamHI restriction enzyme sites. The *OGDH* exon was 147 basepairs (bp) of exonic sequence and 300 bp of intronic sequence flanking on either sides. pSPL3 plasmids were transfected via a 1:4 polyethylenimine (11460630, Fisher Scientific) into HEK293 cells, and after 24 hours, total RNA was extracted using TRIsure (BIO-38033, Bioline) following the manufacturer’s instructions. Reverse-transcriptase polymerase chain reaction (PCR) was performed using the Luna Universal One-Step RT-qPCR Kit (E3005, BioLabs) as per manufacturer instructions using the primers listed in the following. Resulting PCR product was separated on a 1.5% agarose gel for visualization.

pSPL3-F: 5′-TCTGAGTCACCTGGACAACC-3′

pSPL3-R: 5′-ATCTCAGTGGTATTTGTGAGC-3 ′

### Cloning and transgenesis for *Drosophila*

For construction of pUASTattB-dOgdh^S301Y^-Flag and pUASTattB-dOgdh^P193L^-Flag, we performed site-directed mutagenesis using pUASTattB-wild-type dOgdh-Flag as a template^26^ and the following primers.

S301Y-F: 5′-ctgaaggagatcatcgacgtaTATacggagttgggagtggagtcg-3′

S301Y-R: 5 ′-cgactccactcccaactccgtATAtacgtcgatgatctccttcag-3′

P193L-F: 5 ′-gatcgccagttcaagctgcTcagcactaccttcatcggaggcgat-3′

P193L-R: 5′-atcgcctccgatgaaggtagtgctgAgcagcttgaactggcgatc-3′

The constructs were injected into *y*^*1*^*,w*^*1118*^*, ΦC31; PBac(y+ -attP-3B)VK00037* embryos, and transgenic flies were selected.^27^

Generation of flies carrying *U6:3-gRNA*^*dOgdh*^ was described in a previous study.^28^ In brief, a pair of oligonucleotides for guide RNA (gRNA) that target the *dOgdh* exon 2-intron junction were phosphorylated, and cloned into pCFD3.1-w-dU6:3gRNA. The oligonucleotide sequences used for gRNA^dOgdh^ are gRNA-F: 5′-GTCG AGGGATACTTACGGTGTGCA-3′ and gRNA-R: 5′-AAAC TGCACACCGTAAGTATCCCT-3′. pCFD3.1-w-dU6:3gRNA is derived from pCFD3^29^ and was a gift from Simon Bullock (plasmid # 123366 Addgene http://n2t.net/addgene:123366;RID:Addgene_123366). The pCFD3.1-w-dU6:3- gRNA^dOgdh^ construct was injected into *y*^*1*^*,w*^*1118*^*, ΦC31; P(CaryP)attP40* embryos, and transgenic flies were selected.

### *Drosophila* strains and maintenance

We obtained the following *Drosophila* stocks from the Bloomington Drosophila Stock Center at Indiana University—*elav*^*c155*^; *UAS-Cas9.P2*. All flies were maintained at room temperature (21 °C), and crosses were kept at 25 °C.

### Genomic PCR and inference of CRISPR edits analysis

Inference of CRISPR Edits (ICE) analysis (Synthego Performance Analysis, ICE Analysis. 2019. V2.0. Synthego; [9.22.2021].) was performed on fly heads expressing the gRNA^*dOgdh*^ and *dOgdh* complementary DNA (cDNA) carrying wild type or variants P193L to measure the knockout efficiency of the system. Genomic DNA was purified from the fly heads using PureLink Genomic DNA Mini Kit (Invitrogen, Cat # K1820-02) according to the manufacturer’s instructions. To amplify the genomic locus through genomic PCR, we used the primers: dOgdh-gF-1 (5′-CCAACTAATGAGCCCCCAATC-3′) and dOgdh-gR-1 (5′-TTTTTTATTGCAGAGAAGTTTGGTTCCC-3′). For amplification of the *dOgdh* cDNA, we used the primers: pUAST-F2 (5′-CAACTGCAACTACTGAAATCTGC-3′) and dOgdh cDNA #R1 (5′-TGGTAGCTCCTGATGATGGCC-3′). All PCR reactions were executed using One *Taq* Hot Start 2X Master Mix with Standard Buffer (M0484S, NEB). The PCR product was purified using QIAquick PCR Purification Kit (28106, QIAGEN) or QIAquick Gel Extraction Kit (28706, QIAGEN) following the manufacturer’s protocol. Sanger sequencing of the purified PCR product was performed. From the Sanger sequence chromatogram, we were able to proceed with ICE analysis to test the degree of knockout at the *OGDH* genomic locus. To perform ICE analysis for the cDNA, we created an artificial gRNA sequence that was complementary to the end of exon 2 and beginning of exon 3.

### *Drosophila* climbing assay

The method for climbing assay in this study was adapted from Yap et al.^4^ CO_2_ was used as an anesthetic to collect 20 flies into a polystyrene vial containing fresh food. Only males were used because female flies in the same genotype groups presented variation of climbing phenotypes because of various fattening states of ovaries. The flies were allowed to rest for 72 to 96 hours at 20 °C with access to fresh food. The climbing apparatus was created by drawing a line around an empty polystyrene vial at 7.5 cm from the bottom. Flies that were 3-day-old and 7-day-old were transferred to the prepared polystyrene vial without using CO_2_. The top was covered by taping another empty polystyrene vial to the opening, being careful not to completely seal the apparatus. Different polystyrene vials were used for each genotype to prevent any cross-contamination. The flies were allowed to acclimate to the new environment for 10 minutes. To begin the assay, the vials were tapped rapidly 5 times. Climbing behaviors of flies were recorded for 60 seconds. After 60 seconds, the flies were allowed to rest for 10 minutes before repeating the assay. A total of 3 trials were completed. After the completion of the assay, the flies were discarded. The video was quantified by manually watching and analyzing the number of flies that crossed the target line at each time point. The percent of flies that crossed the target line at 60 seconds was averaged for 3 to 4 replicates to create the bar graph.

### *Drosophila* life span assay

CO_2_ was used to anesthetize 20 flies and collect them into a polystyrene vial containing fresh food. Only females were used for life span assay, because culturing only males often resulted in bacterial and fungal contamination, which led to phenotypic variations. The flies were kept at 25 °C over the course of the assay and were checked daily to count the number of flies that survived. The flies were transferred to a new vial with fresh food every 3 to 4 days. The average percentage of flies that survived each day was calculated across 5 to 6 replicates for each genotype to convert the raw data into a line chart.

## Results

### Clinical findings

We identified 4 unrelated individuals (Figure 1C) harboring homozygous variants in *OGDH*, a gene recently implicated in a single family with α-KGDH deficiency, a severe neurodevelopmental disorder.^4^ The 4 individuals included in this study presented with disease within the first year of life. All described individuals shared the clinical features of global developmental delay, hypotonia, metabolic acidosis, and increased serum lactate (Table 1). Additional shared clinical features included dystonia (3/4), microcephaly (3/4), abnormal nasal bridge morphology (3/4), hyperammonemia (3/4), hyperglutaminemia (3/3 who were tested), and elevated α-ketoglutarate in the urine (2/2 who were tested) (Table 1 and Supplemental Table 1).

Individual 1 was born to consanguineous parents of Iranian descent. She was referred to a neurologist at age 4 months because of hypotonia and poor head control. At 7 months she developed myoclonic seizures that were controlled after adrenocorticotropic hormone treatment. By the age of 3½ years, the individual had severe neurodevelopmental delay with hypotonia, dystonia, and ataxia. At the age of 4 years, she was able to sit, crawl, stand, and walk 2 to 3 steps with assistance and her speech was restricted to babbling. Brain magnetic resonance imaging (MRI) at age 1 year and 4 months showed agenesis of the corpus callosum and mild brain atrophy. During infection, episodes of acidotic decompensation and hyperammonemia occurred. Investigations showed metabolic acidosis, increased serum lactate (24.7 mg/dl, reference [ref], <20 mg/dl) and hyperammonaemia (262 μmol/dl, ref <60 μmol/dl). In addition, measurement of urine organic acids showed elevated lactic acid alongside other organic acids, and finally, measurement of plasma amino acids showed elevated glutamine and methionine (Supplemental Table 1).

Individual 2 was born to consanguineous parents of Iranian descent. At age 9 months, he was diagnosed with neurodevelopmental delay. He had onset of seizures at 9½ months, and at 18 months, he had an episode of tonic seizures and epileptic spasms leading to admission to the pediatric intensive care unit. He first sat at age 2 years and began walking at age 2½ years. At age 3 years and 5 months, he had hypotonia, dystonia, ataxia with poor coordination, and dysmorphic features with full cheeks and broad nasal bridge and severe acquired microcephaly with a head circumference measurement of 45 cm (<0.1 percentile). He has severe intellectual disability with a developmental quotient of 65. Speech is restricted to babbling, and he has a diagnosis of autism spectrum disorder. Brain abnormalities on MRI at age 2½ years included systematic atrophy of fronto-temporal lobes, perisylvian atrophy, and mild ventriculomegaly. He had metabolic abnormalities with mild metabolic acidosis, increased serum lactate (26 mg/dl, ref <20 mg/dl), and hyperammonemia (113 μmol/dl, ref <60 μmol/dl) during infection (Supplemental Table 1). Plasma amino acid profile was unavailable from this individual.

Individual 3 was born to consanguineous parents of Ashkenazi Jewish descent. Pediatric visits during the first 5 months showed gross and fine motor developmental delay, bilateral horizontal nystagmus, poor eye fixation and frequent regurgitations with occasional projectile vomiting. At age 5 months, the child had poor head control and was unable to roll from prone to supine, reach out, or grasp objects. She had increased tone in all 4 limbs with bilateral talipes and very brisk tendon reflexes. At a follow-up visit at 34 months, the individual had severe global development delay, poor growth, and acquired microcephaly. She had minor dysmorphic features including bitemporal narrowing and mild depressed nasal bridge. She was able to sit without support, had commando crawling, and immature pincer grasp. She vocalized without clear words and was able to understand simple commands. Brain MRI at 4 months revealed agenesis of the corpus callosum with associated colpocephaly and symmetrical expansion of the temporal horns, severe hypoplasia of the pons, delayed myelination, and deep gray matter signal abnormalities mostly seen in the dentate nuclei, globi pallidi, and in the subthalamic nuclei. On magnetic resonance spectroscopy there was an abnormal doublet peak at 1.3 ppm, suspicious for lactate accumulation. At her initial evaluation, laboratory studies revealed persistent elevation of lactate (5.8 mmol/L, ref <2.2 mmol/L), mild decrease of venous bicarbonate (19.2 mmol/L, ref 21–26 mmol/L) with otherwise normal blood gas, glucose, and ammonia. Plasma amino acids showed elevated glutamine, alanine, and proline and low citrulline. Urine organic acids showed only mild elevations in TCA metabolites (α-ketoglutaric acid, malate, citric acid, and fumarate). She did not tolerate viral illnesses well and at age 17 months, in the setting of viral gastroenteritis, was found to have increased gap metabolic acidosis, high blood lactate (9 mmol/L, ref <2.2 mmol/L), normal ammonia, and low blood glucose level. Plasma amino acids during viral illness revealed the same pattern as seen at the initial evaluation. Urine organic acids showed elevated α-ketoglutaric acid (754 mmol/mol creatine, ref <117 mmol/mol), 2-hydroxyglutaric, fumaric acid, malic acid, and ketosis. She responded well to glucose-containing intravenous fluids with rapid clinical amelioration. Similar metabolic decompensation recurred with other intercurrent illnesses. She was treated with L-carnitine 50 mg/kg/day and thiamine 100 mg twice a day, and subsequently, she had normalization of glucose, glutamine, and organic acid levels.

Individual 4 was born to consanguineous parents of Syrian ethnicity. She presented with hypotonia and weak cry that started in the neonatal period but became more evident at 2 months of age. At first physical examination, severe hypotonia was observed, and she had no head control. She was noted to have frontal bossing, depressed nasal bridge and micrognathia. Brain MRI at 8 months revealed bilateral symmetric hyperintense T2 lesions in the dentate nucleus. Baseline blood lactate was 3.4 mmol/l and elevated to 4.5-6.2 mmol/l during infection. In addition, she presented with hyperammonemia (132 μmol/dl) during severe pneumonia and had elevated urinary α-ketoglutarate (446-691 mmol/mol creatine, ref <117 mmol/mol) and plasma glutamine levels among other plasma amino acids (Supplemental Table 1). She died at age 12 months during an intensive care unit admission for a subsequent episode of severe pneumonia.

### Genetic findings

Owing to the presence of parental consanguinity in all 4 families, and thus the expectation of an autosomal recessive disease, exome sequencing was chosen to search for homozygous rare protein coding variants within affected individuals in families 1, 2, and 4. For family 3 a trio mitochondrial nuclear gene panel was sent to GeneDx following clinical suspicion of a mitochondrial disease. Genetic investigation led to the identification of the suspected novel pathogenic variants within *OGDH* (NM_002541.3) that segregated with disease in the families (Figure 1C and Supplemental Figures 1 and 2). The variants were predicted to occur within, or adjacent to, the TPP binding domain within the OGDH protein (NP_002532.2) (Figure 2A). *OGDH* is a highly constrained gene for both missense and protein loss-of-function variants, with a *z*-score of 4.76 and a pLI score of 1.^16^ Both individuals 1 and 2 shared the same homozygous variant c.890C>A:p.(Ser297Tyr) whereas individual 3 harbored a different homozygous *OGDH* variant c.566C>T:p.(Pro189Leu), and individual 4 presented with a separate homozygous variant c.935G>A:p.(Arg312Lys). All variants were considered to be the most likely cause of disease due to absence in population databases, such as Genome Aggregation Database^16^, and because of damaging predictions from in silico pathogenicity predicting programs such as CADD, PolyPhen, Sorting Intolerant from Tolerant, and REVEL (Supplemental Table 2). The population database Iranome was queried as a population specific database for individuals 1 and 2, and the p.(Ser297Tyr) variant was found to be absent. Iranome is a genetic database containing exome sequencing data from 800 healthy individuals from 8 major ethnic groups in Iran.^15^ All variants have a CADD Phred score of over 20, a PolyPhen prediction of "probably damaging" and REVEL scores ranging from 0.539 to 0.956 (Supplemental Table 2).

### In silico homology modeling and conservation of OGDH variants identified

We investigated the pathogenicity of the 3 OGDH variants in silico. All 3 variants feature substitutions in conserved amino acids (Figure 2B). We used our homology model to predict the impact of the OGDH variants identified in this study (Figure 2C). Our model strongly supported the suspected pathogenicity of the p.(Ser297Tyr) variant in individuals 1 and 2 (Figure 2C). The side chain of tyrosine is considerably larger than the side chain of serine 297 with little space to accommodate the larger aromatic group. Therefore, it is conceivable that this variant leads to structural instability and, potentially, to unfavorable changes in the protein structure, which are propagated to the following β-strand. If so, the impact of the p.(Ser297Tyr) variant on the β-strand could affect the binding of OGDH to its cofactor, TPP. At the end of the β-strand, the histidine 311 makes an ionic interaction with the β-phosphate of the bound TPP that could be interfered with by the aromatic substitution at residue 297. Similarly, our homology model supported the pathogenicity of the p.(Pro189Leu) variant identified in family 3. Proline 189 is located at the OGDH dimer interface and is exposed to a hydrophilic environment (Figure 2C). We speculate that the bulky hydrophobic side chain of the leucine substitution may destabilize the dimer interface. In the case of the p.(Arg312Lys) variant, our homology model showed that the Arg312 side chain makes an ionic interaction with the phosphate group of TPP and is directly involved in cofactor binding. However, we cannot be certain about the pathogenicity of this variant at the protein level because a positively charged lysine side chain could still contribute to TPP binding. The variant c.935G>A:p.(Arg312Lys) is located near a splicing donor site of *OGDH* exon 7, suggesting that the c.935G>A substitution may affect the splicing of *OGDH*. We used the variant splice predictor SpliceAI^20^ to predict whether the 3 variants may impact splicing (Supplemental Table 2). The analysis by SpliceAI predicted that the c.935G>A substitution causes loss of donor site (0.93) whereas the other 2 variants, c.566C>T;p.(Pro189Leu) and c.890C>A:p.(Ser297Tyr), were predicted to have an unlikely splice impact. Taken together, our in silico analysis suggested that the p.(Ser297Tyr) and p.(Pro189Leu) variants would interfere with the structural stability and likely the function of OGDH, and the c.935G>A:p.(Arg312Lys) variant would affect splicing of *OGDH*.

### OGDH^p.(Pro189Leu)^ and OGDH^p.(Ser297Tyr)^ variants cause OGDH protein instability

To understand the impact of the OGDH variants on protein stability in vitro, we transfected HEK293 cells with one of 4 flag-tagged pcDNA3.1 plasmids containing either the OGDH^WT^, OGDH^p.(Pro189Leu)^, OGDH^p.(Ser297Tyr)^ or OGDH^p.(Arg312Lys)^ variants, alongside a GFP plasmid to normalize for transfection efficiency (Figure 2D and Supplemental Figures 3 and 4). Immunoblotting analysis suggested protein instability as a result of the OGDH^p.(Pro189Leu)^ and OGDH^p.(Ser297Tyr)^ variants, with protein levels significantly reduced compared with OGDH^WT^ protein levels, and the production of stable OGDH^p.(Arg312Lys)^ protein. The resultant stable OGDH^p.(Arg312Lys)^ protein is consistent with our prediction that the variant does not affect protein stability. This assay was not designed to inform upon splicing impact because the *OGDH* cDNA sequence harboring variants was inserted into the pcDNA3.1 plasmid; intronic regions were not included and splicing did not occur and so cannot be assessed.

In addition to the HEK293 overexpression experiments, we were also able to investigate OGDH^p.(Ser297Tyr)^ protein stability in fibroblasts collected from individual 1. The mitochondrial network in fibroblasts from individual 1 appeared normal (Supplemental Figure 5). We decided to assess the stability of the OGDH protein produced by individual 1 fibroblasts by inhibiting translation with cycloheximide. Endogenous OGDH protein levels were measured in individual 1 fibroblasts against control fibroblasts and normalized to TOM20 protein levels to account for mitochondrial mass. The OGDH protein level with no treatment (0 μg/mL) was seen not to be significantly reduced in the individual 1 cells (Figure 2E and Supplemental Figures 6 and 4). We found that treatment with cycloheximide revealed a higher rate of protein turnover in individual 1 fibroblast cells with the p.(Ser297Tyr) variant than that of wild-type OGDH. These data are in line with the predictions with the homology modeling (Figure 2C) and HEK293 overexpression data (Figure 2D) in supporting OGDH^p.(Ser297Tyr)^ as a potential protein instability variant. Unfortunately, primary fibroblasts were unavailable for the other individuals in this study.

To confirm the suspected splicing defect of the c.935G>A variant, we performed an exon-trapping mini-gene assay with the pSPL3 vector (Figure 2F and G). *OGDH* exon 7 was introduced into the pSPL3 splicing vector, with or without the c.935G>A variant change, and transfected into HEK293 cells, and RNA was extracted and converted to cDNA. The cDNA band size informs on the splicing impact of the c.935G>A variant. Normal splicing results in a band of 410 bp, as is depicted in the upper half of Figure 2F, and can be seen to be produced from the OGDH^WT^ plasmid (Figure 2G). The OGDH^c.935G>A^ plasmid lacked the normal 410 bp product and produced a 270 bp band (Figure 2G), which was confirmed, through Sanger sequencing, to be a result of complete skipping of OGDH exon 7. This is depicted in the lower half of Figure 2F. Some additional alternatively spliced mRNA products were produced by the OGDH^c.935G>A^ plasmid; however, levels were too low to allow for Sanger sequencing to determine their sequence. We further predicted that, after the deletion of exon 7, the protein remains in-frame. Without the c.935G>A variant, arginine (AGG) is formed between the last 2 nucleotides of exon 6 (c.787-788) and the G beginning at exon 7 (c.789). When the c.935G>A variant is present and exon 7 is deleted, we predicted that the AG at the end of exon 6 (c.787-788) will form a codon with the first nucleotide (c.934) of exon 8 instead resulting in an AGA, thereby still producing arginine. Therefore, the variant consequence can be described at the protein level as p.(Phe264_Arg312del).

### Functional analysis of OGDH variants in *Drosophila*

**Figure 3:**
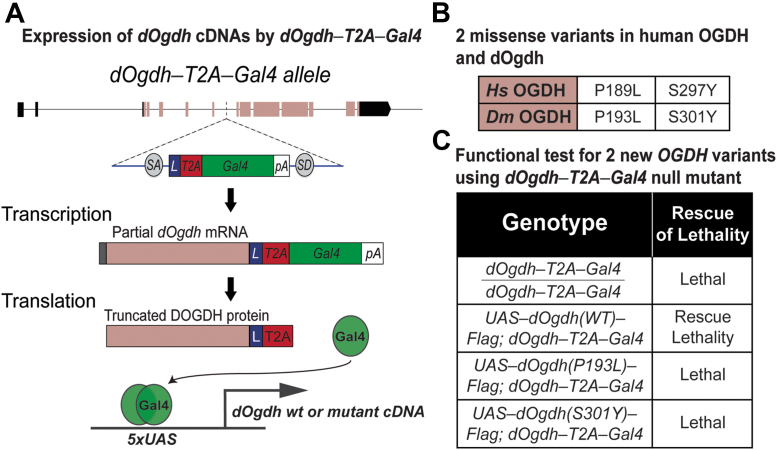
**Novel missense variants in *dOgdh* are lethal in a *Drosophila* model.** A. A schematic of the expression of Gal4 from *dOgdh-T2A-Gal4* allele and Gal4/UAS-mediated expression of wild-type or mutant *dOgdh* cDNA. B. A table displaying the missense variants in human OGDH and their homologous variants in *Drosophila Ogdh* (*dOgdh*). C. The lethality caused by *dOgdh-T2A-Gal4* null allele was rescued by *dOgdh* wild-type cDNA expression. Expression of *dOgdh* carrying p.(Pro193Leu) or p.(Ser301Tyr) failed to rescue the lethality of *dOgdh* null mutant. cDNA, complementary DNA; gRNA, guide RNA; OGDH, oxoglutarate dehydrogenase; UAS, upstream activating sequence.

To determine whether the *OGDH* variants identified from probands have functional effects in vivo, we used *Drosophila* as a genetic model. In our previous study, we created a null mutant of *Drosophila Ogdh* (*dOgdh*)—*dOgdh-T2A-Gal4* in which a DNA cassette carrying splicing acceptor, T2A-Gal4 cDNA, and poly A sequence was inserted into the coding intron of *dOgdh* using recombination-mediated cassette exchange (Figure 3A).^26^ *dOgdh-T2A-Gal4* mutant expresses partial *dOgdh* mRNA and protein, resulting in loss of function of *dOgdh*, and Gal4 is expressed under the control of the cis-regulatory elements of *dOgdh* (Figure 3A). Gal4 binds to upstream activating sequence (UAS) that activates expression of genes downstream of UAS (Figure 3A). We previously showed that *dOgdh* is ubiquitously expressed,^4^ and expression of wild-type *dOgdh* cDNA rescued lethality caused by loss of d*Ogdh*, but *dOgdh* cDNA carrying a homologous variant in human *OGDHL* variant (p.Ser793Leu) could not.^26^ This prior study demonstrated that *dOgdh-T2A-Gal4* mutant is an excellent tool for in vivo functional study of pathogenic candidate variants in the human *OGDH* paralogs. Hence, we sought to determine the functional effects of p.(Ser297Tyr) and p.(Pro189Leu) variants in *OGDH* using *dOgdh-T2A-Gal4* mutant. To this end, we generated transgenic flies carrying *dOgdh* cDNA with homologous variants to the human *OGDH* variants under the control of UAS (*dOgdh*^*P193L*^ and *dOgdh*^*S301Y*^) (Figure 3B). We found that expression of *dOgdh*^*P193L*^ or *dOgdh*^*S301Y*^ failed to rescue the lethality caused by *dOgdh* loss. Hence, the results indicate that *dOgdh*^*P193L*^ and *dOgdh*^*S301Y*^ are loss-of-function alleles.

**Figure 4:**
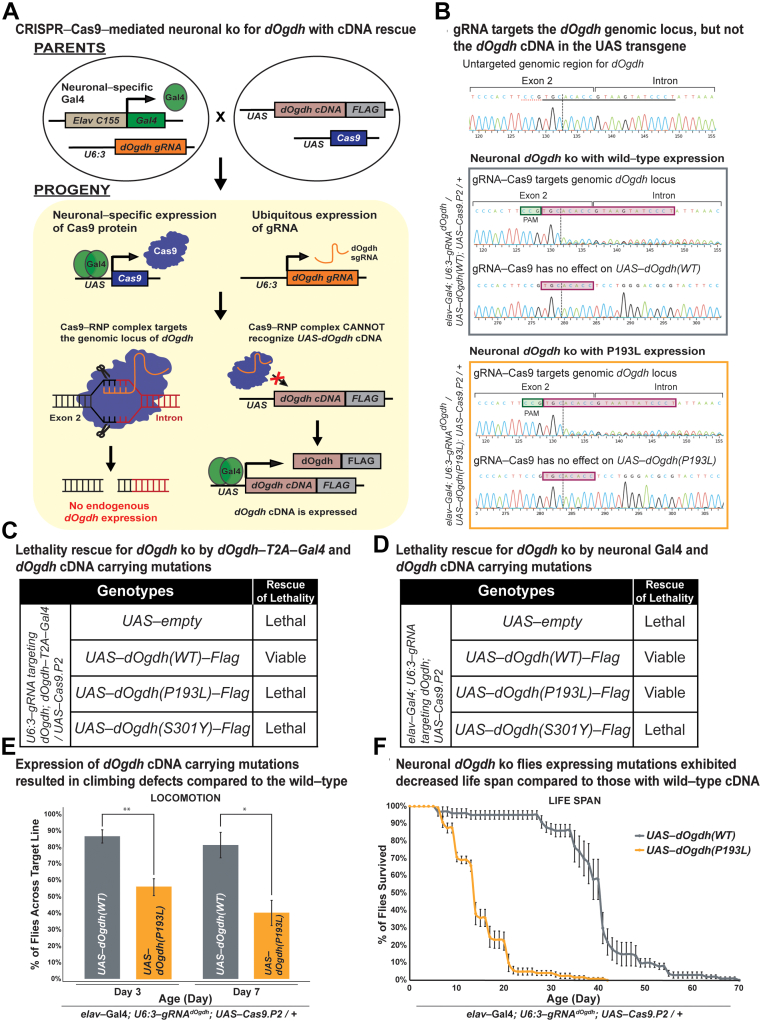
**CRISPR-Cas9-mediated neuronal knockout of *dOgdh* reveals different allelic strengths in 2 missense variants.** A. Schematic illustrating neuronal-specific knockout of *dOgdh* and cDNA rescue by employing sgRNA/Cas9-RNP that specifically targets the exon-intron junction of *dOgdh* genomic locus but does not target *UAS*-*dOgdh* cDNA transgene in *Drosophila*. B. Sanger sequencing chromatograms for a gRNA^dOgdh^ target region at the *dOgdh* genomic locus and *UAS-dOgdh* cDNA transgene region. The first chromatogram was obtained from flies that carry *elav*^*C155*^*-Gal4*; *U6:3-gRNA*^*dOgdh*^ but not *UAS-Cas9.P2*. The gray box includes chromatograms for flies having *elav*^*C155*^*-Gal4*, *U6:3-gRNA*^*dOgdh*^, *UAS-Cas9.P2*, and *UAS-dOgdh(WT).* The orange box includes chromatograms for flies carrying *elav*^*C155*^*-Gal4*, *U6:3-gRNA*^*dOgdh*^, *UAS-Cas9.P2*, and *UAS-dOgdh(P193L).* The pink boxes indicate the gRNA^dOgdh^ target site at the genomic locus. The green boxes indicate the PAM sequence of the gRNA^dOgdh^. C. The lethality caused by *dOgdh* knockout in *dOgdh*-expressing cells (*dOgdh-T2A-Gal4*) was rescued by wild-type *dOgdh* cDNA but not by cDNA carrying variants p.(Pro193Leu) or p.(Ser301Tyr). D. Lethality due to neuronal-specific knockout of *dOgdh* was rescued by expression of wild-type *dOgdh* cDNA as well as the cDNA encoding p.(Pro193Leu) but not by the expression of *dOgdh* cDNA carrying p.(Ser301Tyr). E. Flies carrying neuronal *dOgdh* knockout with expression of p.(Pro193Leu) exhibited climbing defects at day 3 to day 7. Error bars indicate SEM. *P* values were calculated by the *t* test. ∗*P* < .05, ∗∗*P* < .01. F. Flies carrying neuronal *dOgdh* knockout with p.(Pro193Leu) expression exhibited shorter lifespan than those with the wild-type *dOgdh* expression. cDNA, complementary DNA; gRNA, guide RNA; UAS, upstream activating sequence.

Although the *dOgdh-T2A-Gal4* mutant allele is suitable to test effects of variants in all tissues and cells that express *dOgdh*, this allele cannot determine the effect of variants in a tissue-specific manner. To determine the effects of the variants in a tissue-specific manner, we decided to use a recently-developed CRISPR-Cas9-mediated system that permits both tissue-specific *dOgdh* knockout and rescue by *dOgdh* cDNA.^28^^,^^31^ We designed a gRNA that is complementary to the exon-intron junction of *dOgdh* and created transgenic flies carrying this gRNA under the control of U6:3 promoter (*U6:3-gRNA*^*dOgdh*^). The U6:3 promoter leads to ubiquitous expression of the *gRNA*^*dOgdh*^. Expression of *UAS-Cas9.P2* by different Gal4 drivers confer the tissue specificity. Our strategy is illustrated in Figure 4A. To validate the system, first, we tested *dOgdh* knockout using *dOgdh-T2A-Gal4* driver. We found that the *dOgdh* knockout from Cas9.P2 expression by *dOgdh-T2A-Gal4* resulted in lethality, which was rescued by wild-type *dOgdh* cDNA (*dOgdh*^*WT*^) expression. In contrast, the lethality was not rescued by *dOgdh*^*P193L*^ or *dOgdh*^*S301Y*^ expression (Figure 4C). The data are consistent with those from the *dOgdh-T2A-Gal4* null mutant background (Figure 3C). We then sought to determine the effects of the variants in the nervous system by using a pan-neuronal Gal4 driver (*elav*^*C155*^*-Gal4*). Neuronal *dOgdh* knockout by *elav*^*C155*^*-Gal4* caused lethality, which was rescued by expression of *dOgdh*^*WT*^ (Figure 4D). By performing Sanger sequencing of fly heads, we found that gRNA^dOgdh^ -Cas9.P2 led to insertion and deletion (indel) variants at the gRNA target region of the *dOgdh* genomic locus but not UAS-*dOgdh*^*WT or P193L*^ transgenes (*elav*^*C155*^*-Gal4; gRNA*^*dOgdh*^*/UAS-dOgdh*^*WT(or P193L)*^*; UAS-Cas9:P2/+*) (Figure 4B). ICE analysis of the Sanger chromatograms confirmed that gRNA^dOgdh^ -Cas9.P2 caused the effective knockout (KO) of *dOgdh* (KO score, 34∼36), but does not have any effect on *UAS-dOgdh cDNA* transgenes (KO score, 0) (Figure 4B). We found that expression of *dOgdh*^*S301Y*^ failed to rescue the lethality caused by neuronal knockout of *dOgdh* (Figure 4D). In contrast, expression of *dOgdh*^*P193L*^ rescued the lethality caused by neuronal *dOgdh* knockout (Figure 4D), but these flies exhibited defects of locomotion and short life span compared with wild-type control flies (Figure 4E and 4F). Hence, the results indicated that *dOgdh*^*S301Y*^ is a severe loss-of-function mutant, and *dOgdh*^*P193L*^ is a hypomorph in the nervous system. Taken together, *Drosophila* functional studies from the *dOgdh* null mutant and neuronal-specific knockout by CRISPR-Cas9 editing support the pathogenicity of p.(Pro189Leu) and p.(Ser297Tyr) in OGDH in humans.

## Discussion

In this study, we have identified 3 novel pathogenic OGDH variants in 4 unrelated families. Through in silico, in vitro, and in vivo analyses, we have confirmed the pathogenicity of the variants p.(Pro189Leu), p.(Ser297Tyr), and p.(Phe264_Arg312del). Recently, a single family was published as harboring a suspected pathogenic OGDH variant p.(Asn320Ser) in 2 siblings exhibiting a severe neurodevelopmental disease.^4^ The siblings shared clinical symptoms with the 4 individuals with pathogenic OGDH variants in this study (Table 1) and, validated by experimental work demonstrating pathogenicity of variants, we confirm recessive OGDH variants as a cause of neurodevelopmental disease.

The hallmark clinical features observed in this study are global developmental delay, hypotonia, dystonia, microcephaly, abnormal nasal bridge morphology, metabolic acidosis, mild hyperammonemia with much higher than expected plasma glutamine level, increased serum lactate, and elevated α-ketoglutarate concentration in the urine (Table 1 and Supplemental Table 1). The clinical characteristics of global developmental delay, hypotonia, dystonia and increased serum lactate are shared with the published sibling pair with the suspected pathogenic OGDH variant p.(Asn320Ser) (Table 1).^4^ The disease phenotypes observed in the 4 individuals in this study and the published siblings with OGDH deficiency overlap with the reported clinical features of α-KGDH deficiency reported in the literature.^10^^,^^11^^,^^32^, ^33^, ^34^ In addition, the MRI findings and elevated lactate are comparable with the Leigh syndrome spectrum in 3 of the individuals reported.

The OGDH variants p.(Pro189Leu) (individual 3) and p.(Ser297Tyr) (individuals 1 and 2) are considered to be highly deleterious missense variants. In silico and homology modeling predictions of the p.(Pro189Leu) and p.(Ser297Tyr) variants indicated pathogenicity and a potential disease mechanism due to protein instability (Figure 2C and Supplemental Table 2). To investigate this in vitro, both flag-tagged variants were overexpressed in HEK293 cells and found to be in significantly decreased amounts compared with wild-type OGDH-FLAG (Figure 2D). However, there are limitations that must be considered when interpreting these results; the HEK293 cells were transfected with 2 separate plasmids, a pcDNA3.1 and a GFP construct, for normalization, and it has been assumed that the transfection rate of each plasmid is equal, but this may not be the case. We further investigated the impact of the p.(Ser297Tyr) variant within fibroblasts for individual 1. Cycloheximide, a translation inhibitor, was used to observe OGDH protein degradation more accurately over a 24-hour period. We did observe the OGDH^p.(Ser297Tyr)^ protein undergo a higher rate of protein turnover than OGDH^WT^ (Figure 2E), suggesting that the OGDH^p.(Ser297Tyr)^ protein is less stable and more vulnerable to cellular degradation, and thus, provide this as further support to the pathogenicity of the p.(Ser297Tyr) variant. Unfortunately, fibroblasts were not available for the other individuals from this study. The data suggests that protein instability is the most likely mechanism leading to a reduction in the levels of OGDH-FLAG protein carrying the p.(Ser297Tyr) and p.(Pro189Leu) variants in the HEK293 overexpression assay; although, this could also be because of variant impact upon RNA expression or protein translation. However, because we observe increased degradation of OGDH protein with the p.(Ser297Tyr) variant in individual 1 fibroblasts, it is likely the mechanism impacting protein level is decreased protein stability.

To determine the functional effects of the p.(Pro189Leu) and p.(Ser297Tyr) variants in vivo, we used a *Drosophila* model in which proline 189 and serine 297 residues in OGDH are conserved (Figures 2B and 3B). Similar to human OGDH, *dOgdh*, its *Drosophila* homolog, is ubiquitously expressed.^4^ The experiments involving *dOgdh* null mutant (*dOgdh-T2A-Gal4*) support the pathogenicity of p.(Pro189Leu) and p.(Ser297Tyr) because fly cDNA carrying the homologous variants failed to rescue lethality caused by loss of *dOgdh* (Figure 3C)*.* In addition, the CRISPR/Cas9-mediated knockout with cDNA rescue system enabled us to determine the effects of the variants in a tissue-specific manner. Importantly, we found that in neurons, *dOgdh*^*S301Y*^ (human p.Ser297Tyr) completely failed to rescue *dOgdh* loss, whereas *dOgdh*^*P193L*^ (human p.Pro189Leu) partially rescued the phenotypes caused by neuronal *dOgdh* loss (Figure 4D and F). The results suggest that p.(Ser297Tyr) is a severe loss-of-function allele and p.(Pro189Leu) is a hypomorph, which is consistent with the high CADD score (p.[Ser297Tyr], 29.04; p.[Pro189Leu], 24.8). Given that p.(Ser297Tyr) affects TPP binding based on the homology modeling and that the protein levels of OGDH^p.(Ser297Tyr)^ are comparable to those of OGDH^p.(Pro189Leu)^ (Figure 2D), we surmise that p.(Ser297Tyr) may affect the enzyme activity of OGDH in addition to the protein stability (Figure 2E). Given the lethality of a *dOgdh* knockout in *Drosophila*, we predict that a total loss of OGDH is incompatible with life.

The homozygous variant c.935G>A (individual 4) differs from the other variants within the study because we predicted it to impact splicing. The G to A transition occurs within the 5′ splice donor site, 1 base upstream of the highly conserved intronic GT nucleotide pair. SpliceAI^20^ predicted the variant to likely result in loss of donor splice site (0.93) (Supplemental Table 2). We tested this in a mini-gene splicing assay with the SPL3 plasmid and found that the c.935G>A variant is responsible for a total loss of *OGDH* exon 7 (Figure 2F and G). Exon 7 consists of 49 amino acids from position 263 to 312 within the OGDH protein. This loss occurs within the TPP binding domain (Figure 2A), and therefore, we hypothesize that one potential outcome of this variant could be an impairment of TPP binding. We further predicted that the c.935G>A variant results in an in-frame OGDH protein consisting of 974 amino acids without any secondary missense variant. Within the wild-type OGDH sequence, the AG nucleotide pair at the end of exon 6 (c.787-788) combines with the first nucleotide G in exon 7 (c.789) to form arginine. With the loss of exon 7 due to the c.935G>A splice variant, we suspect that the AG nucleotides at position c.787-788 will instead form a codon with the first base of exon 8, which is adenine (c.934). This new codon, AGA, retains the formation of arginine and keeps the protein in-frame. Hence, the impact of the c.935G>A is predicted to be a loss of exon 7 only. Additional bands of around 350 bp and 450 bp are seen as a result of the c.935G>A variant (Figure 2G). Unfortunately, Sanger sequencing of these bands failed. However, we suspect that the c.935G>A variant activates cryptic splice sites leading to these alternatively spliced products. Following our analysis and predictions, the c.935G>A variant can be described at the protein level as p.(Phe264_Arg312del).

OGDH requires the cofactor TPP, an important coenzyme for multiple protein-catalyzed cellular reactions, and all 4 variants appear to cluster within or bordering the TPP binding domain (Figure 2A). Thiamine is already considered as a management option for individuals with metabolic disorders such as pyruvate dehydrogenase complex deficiency (OMIM 312170) and maple syrup urine disease (OMIM 248600). Individuals with maple syrup urine disease with residual enzyme activity of at least 5% were found to have a therapeutic response to thiamine over a 4 week treatment period.^35^ Similarly, individuals with pyruvate dehydrogenase complex deficiency have been found to respond to thiamine treatment.^36^, ^37^, ^38^ Moreover, 2 individuals with pathogenic variants located within the TPP binding domain of the pyruvate dehydrogenase E1α subunit (*PDHA1*) were successfully treated with thiamine, which rescued the decreased affinity of the aberrant E1α subunit to TPP.^39^ Given the location of the variants identified in this study to either within or in close proximity to the TPP binding domain, thiamine treatment should be considered to potentially increase TPP binding affinity. Individual 3 in this study is currently being treated with thiamine 100 mg twice a day and is reported to have stabilized glucose, glutamine, and organic acid measurements. We therefore suggest that high dose thiamine treatment should be considered for individuals with OGDH deficiency.

Owing to the central role of the TCA cycle in cellular metabolism, defects can lead to a range of disorders and impact other critical cellular processes (Figure 1A). Observed in our study is the increase of ammonia, much higher than expected glutamine levels in the blood, increased serum lactate, and metabolic acidosis (Table 1). α-Ketoglutarate holds a critical role in glutamine metabolism; glutamine can be metabolized to α-ketoglutarate via 2 pathways, glutaminolysis and glutaminase (GLS) II^40^ (Supplemental Figure 7). Glutaminolysis converts glutamine to glutamate and ammonia before glutamate is reversibly converted to α-ketoglutarate. The GLS II pathway leads to the transamination of glutamine to α-ketoglutaramate, which is hydrolyzed to α-ketoglutarate and ammonia. Moreover, α-ketoglutarate can be converted back to glutamate and glutamine via glutamate dehydrogenase and glutamine synthetase (Supplemental Figure 7). Pathogenic *OGDH* variants resulting in deficiency of the α-KGDH complex likely result in a block of the TCA cycle at the conversion of α-ketoglutarate to succinyl-CoA, leading to the elevated urinary α-ketoglutarate levels in OGDH deficient individuals (Table 1). A build-up of α-ketoglutarate could thus lead to reversal of the glutaminolysis and GLS reactions and thus explain why increased levels of glutamine and ammonia are observed in individuals with OGDH deficiency.

Critical to the support of *OGDH* variants as pathogenic is the prior association of the α-KGDH complex, of which OGDH is a component, together with DLD and DLST, with disease.^10^^,^^11^ Moreover, the loss of nardilysin (*NRDC*; OMIM 602651), that encodes a mitochondrial cochaperone involved in OGDH folding, has been shown in *Drosophila* to lead to decreased levels of *dOGDH* and neurodegeneration by affecting the mammalian target of rapamycin activity and decreasing autophagy.^26^ Furthermore, biallelic variants in the 2-oxoglutarate dehydrogenase-like (OGDHL) protein, which is also essential to the conversion of α-ketoglutarate to succinyl-CoA, have recently been associated with neurodevelopmental disease.^28^ Although OGDH and OGDHL catalyze the same reaction, the finding that variants in both genes lead to a neurodevelopmental disease suggest that they are not functionally redundant and/or that they have distinct expression patterns in humans. Indeed, OGDHL expression is localized mainly in the brain whereas OGDH is ubiquitously expressed in human tissue.^41^ Furthermore, we previously demonstrated that *OGDHL* knockout in human neuronal cells leads to a substantial decrease of oxygen consumption rates and ATP production compared with the control cells,^28^ indicating that both enzymes are required for optimal mitochondrial metabolism in human neurons. Rat brain extract having both OGDH and OGDHL showed a biphasic substrate (α-ketoglutarate) saturation kinetics.^42^ Hence, we anticipate that OGDH and OGDHL have different binding affinities to α-ketoglutarate, which enables the brain to run the TCA cycle using a wide range of α-ketoglutarate concentration in neurons.^28^

A recent publication has described for the first time the noncanonical TCA cycle.^43^ In contrast to the traditional TCA cycle (Figure 1A), the noncanonical cycle transports citrate out of the mitochondria into the cytosol where it undergoes conversion to malate that is transported back into the mitochondria to complete the cycle enabling it to begin again. The purpose of this alternative cycle was demonstrated to be in response to mammalian cell state. The discovery of this noncanonical cycle is of particular interest in metabolic diseases originating from defective enzymes within the traditional TCA cycle. Given that the noncanonical TCA cycle functions without dependence on most traditional TCA cycle complexes, including the α-KGDH complex, it would be interesting to assess whether its function is altered in response to aberrations in the traditional TCA cycle, such as those present in the individuals in this study.

Finally, genes implicated in autosomal dominant and x-linked diseases are generally highly constrained and intolerant to both protein truncating and missense variation.^44^ Although autosomal recessive genes tend to be much less constrained, *OGDH* is highly constrained for both protein truncating (pLI = 1) and missense (*z*-score = 4.76) variation. However, a substantial proportion of autosomal recessive genes are highly constrained, and therefore high constraint metrics should not necessarily be used to exclude genes as candidates causing autosomal recessive disease.^45^

In summary, we confirm the pathogenicity of *OGDH* variants through the identification of 3 additional homozygous pathogenic variants. We provide a clear phenotype-genotype correlation with in silico, in vitro, and in vivo evidence to support pathogenic variants in *OGDH* leading to a neurodevelopmental disease characterized by global developmental delay, movement disorder, and metabolic aberrations.

## Data Availability

Data are available upon request.

## Acknowledgments

We thank the families for their participation in this study.

## Funding

W.H.Y. is supported by the National Institute of Neurological Disorders and Stroke (5R01 NS121298-02) of the National Institutes of Health (NIH), Presbyterian Health Foundation (4411-09-10-0), and Oklahoma Center for Adult Stem Cell Research (OCASCR 221009). W.H.Y. was also supported by the National Institute of General Medical Sciences (5 P20 GM103636-09) of the NIH and Presbyterian Health Foundation (4411-05-07-0). S.L. is supported by a grant from the NIH (R01-GM142143). E.W. is supported by the Medical Research Council (MR/N013638/1). H.H. is funded by the Medical Research Council (MR/S01165X/1, MR/S005021/1, G0601943), The NIHR University College London Hospitals Biomedical Research Centre, Rosetrees Trust, Ataxia UK, Multiple System Atrophy Trust, Brain Research UK, Sparks GOSH Charity, Muscular Dystrophy UK, and Muscular Dystrophy Association.

## Author Information

Conceptualization, Methodology and Supervision: C.J.C., W.H.Y.; Data Curation: A.M.P.; Investigation: E.F.W., E.G.K., M.C., H.P., S.L., D.B., M.K., M.B.T., P.N.T., R.S.B., I.S., H.Y., S.R., R.M., C.J.C.; Writing-original draft: E.F.W., M.C., W.H.Y., C.J.C., E.I.; Writing-review and editing: E.F.W., M.C., E.G.K., H.P., D.B., M.K., R.D.G., M.B.T., P.N.T., R.S.B., I.S., H.Y., H.Z.E., S.L., Y.J., A.M.P., H.H., E.I., S.R., R.M., W.H.Y., C.J.C.; Visualization: E.F.W.

## Ethics Declaration

Ethical approval was obtained from Mashhad University of Medical Sciences Research Ethics Committee (IR.MUMS.REC.1395.40) for individuals 1 and 2 and all subsequent institutions providing data involved in the study obtained local research ethics committee approval. Informed written consent was obtained for all individuals involved in the study. For individual 3, sequencing was performed on a diagnostic basis and informed consent was provided for publication. All clinical data are de-identified. Our study adheres to the principles set out by the Declaration of Helsinki.

## Conflict of Interest

H.Z.E. and H.Y. are employees of GeneDx, LLC. R.D.G. is a consultant for Minovia Therapeutics.

All other authors declare no conflicts of interest.
