# Supplementary material for: Biallelic variants in OGDH encoding oxoglutarate dehydrogenase lead to a neurodevelopmental disorder characterized by global developmental delay, movement disorder, and metabolic abnormalities
Source: Genet Med. 2023 Feb;25(2):100332. doi: 10.1016/j.gim.2022.11.001 (PMC9905285; doi:10.1016/j.gim.2022.11.001)
Supplement: Supplemental Figure [file mmc2.pdf]

# Supplemental Data

**Supplementary Table 1. Additional clinical findings in individuals with homozygous *OGDH* pathogenic variants (separate excel file).**

**Supplementary Table 2. Variant information for the three *OGDH* variants presented in this study, in addition to the already reported homozygous variant p.Asn320Ser (4).**

| | **ID** | **Individual 1** | **Individual 2** | **Individual 3** | **Individual 4** | **Individual 5 (Yap 2021)** | **Inividual 6 (Yap 2021)** |
|---|---|---|---|---|---|---|---|
| | **Variant** | c.890C>A p.(Ser297Tyr) | | c.566C>T p.(Pro189Leu) | c.935G>A p.(Arg312Lys)/ p.(Phe264_Arg 312del) | c.959A>G p.(Asn320Ser) | |
| | **Homozygous** | Yes | | Yes | Yes | Yes | |
| | **Transcript** | NM_002541 | | NM_002541 | NM_002541 | NM_002541 | |
| | **Ref build** | GRCh38 | | GRCh38 | GRCh38 | GRCh37 | |
| | **Location** | 7:44674512 | | 7:44666784 | 7:44674557 | 7:44714800 | |
| Population Frequencies | **gnomAD** | 0 | | 0 | 0 | 0 | |
| | **Iranome** | 0 | | 0 | 0 | 0 | |
| | **GME TP** | 0 | | 0 | 0 | 0 | |
| Predictive Scores | **CADD Phred** | 29.4 | | 24.8 | 36 | 24.7 | |
| | **SIFT Score** | 0 | | 0.01 | 0 | 0 | |
| | **SIFT Prediction** | damaging | | damaging | damaging | damaging | |
| | **Polyphen** | probably damaging (1.00) | | probably damaging (0.997) | probably damaging (1.00) | probably damaging (1.00) | |
| | **SpliceAI** | Unlikely splice impact | | Unlikely splice impact | Donor loss 0.93 | Unlikely splice impact | |
| | **REVEL** | 0.956 | | 0.539 | 0.899 | 0.819 | |
| | **ACMG** | Likely pathogenic (PM2 PP3 PS3 PP2) | | Likely pathogenic (PM2 PP3 PS3 PP2) | Pathogenic (PVS1 PS3 PM2 PP2) | | |

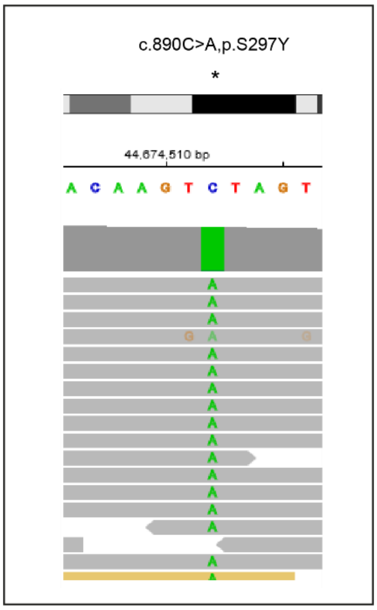


**Supplementary Figure 1 | Integrative Genomics Viewer (IGV) image of sequencing data for individual 1 p.(Ser297Tyr), aligned to GRCh38 human reference genome.**

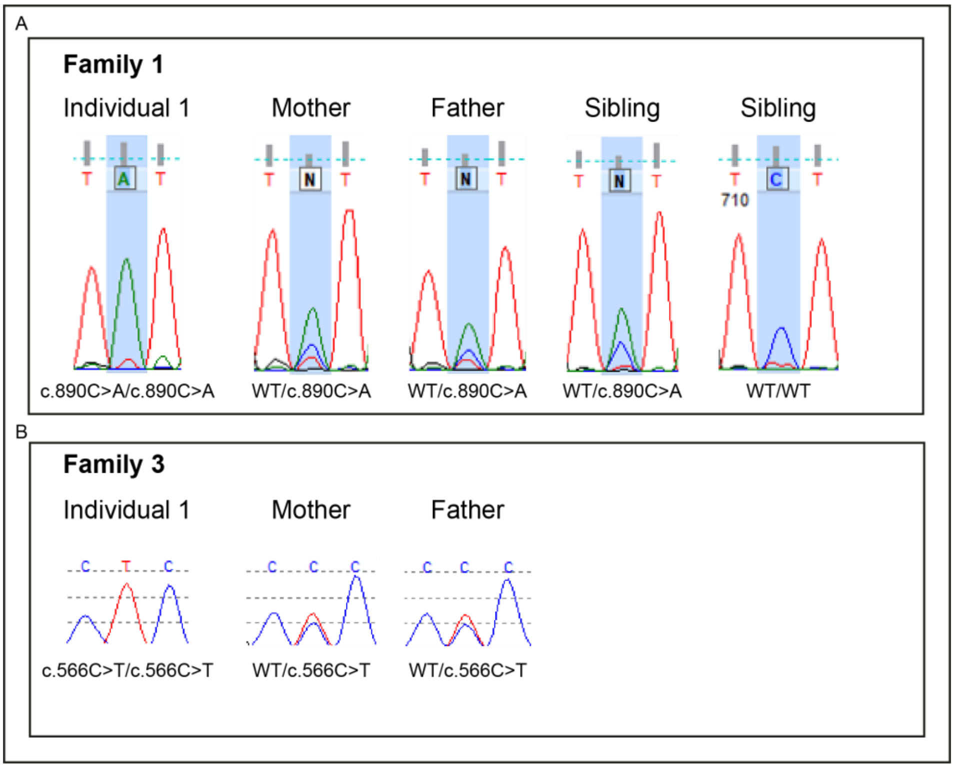


**Supplementary Figure 2 | Segregation analysis.** (A) Segregation data for family 1 harbouring the p.(Ser297Tyr) variant. (B) Segregation data from family 3 harbouring the p.(Pro189Leu) variant.

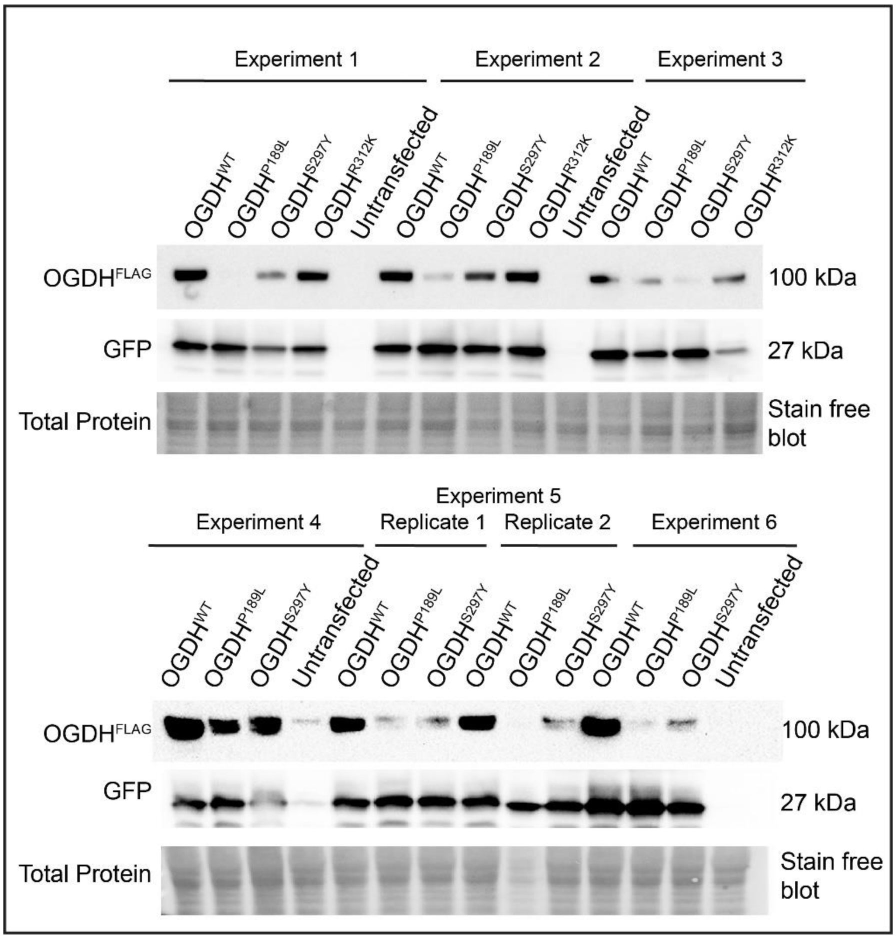


**Supplementary Figure 3 | Replicates of HEK293 transfection with pcDNA3.1 containing a flag tagged OGDH variant of interest.** Transfection of either pcDNA3.1-OGDH$^{WT}$-FLAG, pcDNA3.1-OGDH$^{p.(Pro189Leu)}$-FLAG, pcDNA3.1-OGDH$^{p.(Ser297Tyr)}$-FLAG, pcDNA3.1-OGDH$^{p.(Arg312Lys)}$-FLAG or transfected with vector only was performed, with GFP and total protein staining for normalisation. The data shown (excluding experiment 5 replicate 2) was used for statistical analysis presented in Figure 2D.

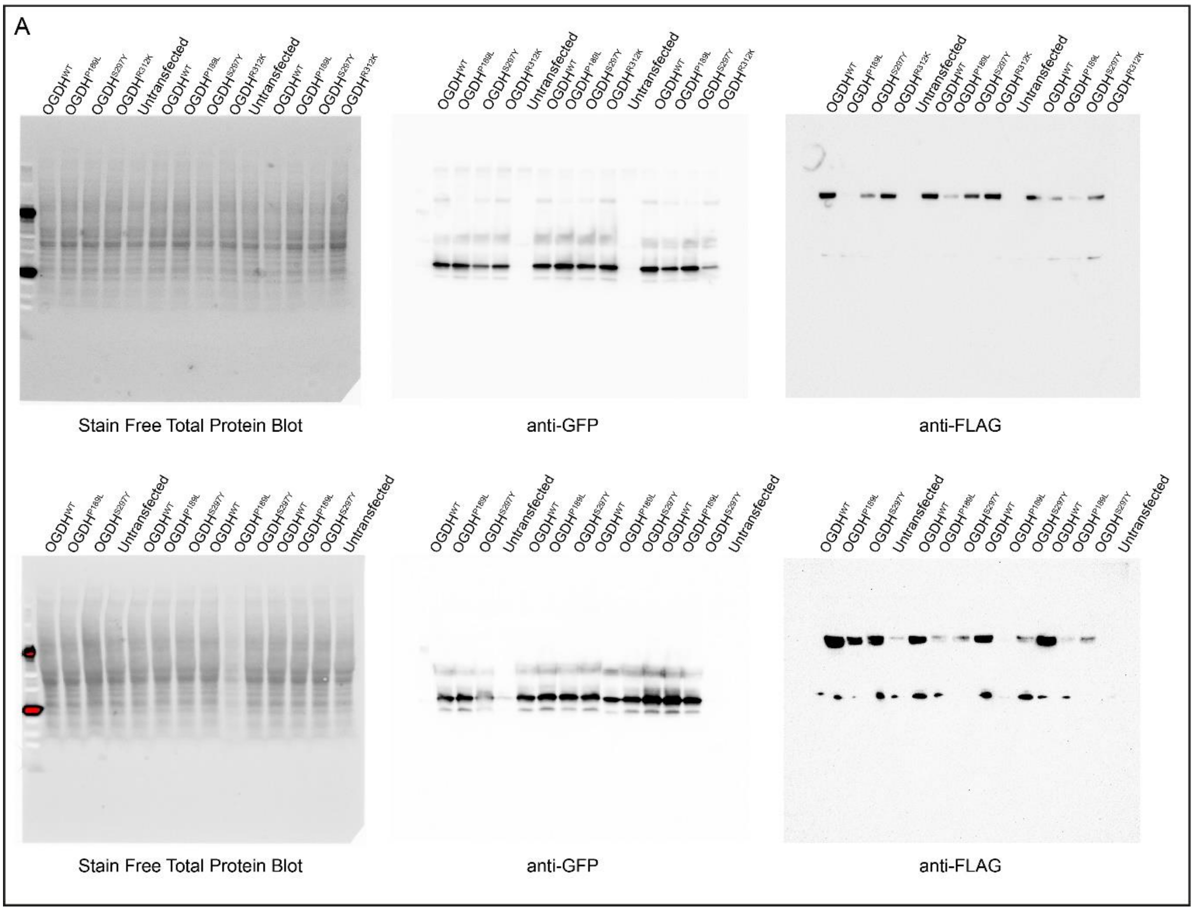

A
OGDH$^{WT}$ OGDH$^{P189L}$ OGDH$^{S297Y}$ OGDH$^{R312K}$ Untransfected OGDH$^{WT}$ OGDH$^{P189L}$ OGDH$^{S297Y}$ OGDH$^{R312K}$ Untransfected OGDH$^{WT}$ OGDH$^{P189L}$ OGDH$^{S297Y}$ OGDH$^{R312K}$
Stain Free Total Protein Blot
OGDH$^{WT}$ OGDH$^{P189L}$ OGDH$^{S297Y}$ OGDH$^{R312K}$ Untransfected OGDH$^{WT}$ OGDH$^{P189L}$ OGDH$^{S297Y}$ OGDH$^{R312K}$ Untransfected OGDH$^{WT}$ OGDH$^{P189L}$ OGDH$^{S297Y}$ OGDH$^{R312K}$
anti-GFP
OGDH$^{WT}$ OGDH$^{P189L}$ OGDH$^{S297Y}$ OGDH$^{R312K}$ Untransfected OGDH$^{WT}$ OGDH$^{P189L}$ OGDH$^{S297Y}$ OGDH$^{R312K}$ Untransfected OGDH$^{WT}$ OGDH$^{P189L}$ OGDH$^{S297Y}$ OGDH$^{R312K}$
anti-FLAG
OGDH$^{WT}$ OGDH$^{P189L}$ OGDH$^{S297Y}$ Untransfected OGDH$^{WT}$ OGDH$^{P189L}$ OGDH$^{S297Y}$ OGDH$^{WT}$ OGDH$^{P189L}$ OGDH$^{S297Y}$ OGDH$^{WT}$ OGDH$^{P189L}$ OGDH$^{S297Y}$ Untransfected
Stain Free Total Protein Blot
OGDH$^{WT}$ OGDH$^{P189L}$ OGDH$^{S297Y}$ Untransfected OGDH$^{WT}$ OGDH$^{P189L}$ OGDH$^{S297Y}$ OGDH$^{WT}$ OGDH$^{P189L}$ OGDH$^{S297Y}$ OGDH$^{WT}$ OGDH$^{P189L}$ OGDH$^{S297Y}$ Untransfected
anti-GFP
OGDH$^{WT}$ OGDH$^{P189L}$ OGDH$^{S297Y}$ Untransfected OGDH$^{WT}$ OGDH$^{P189L}$ OGDH$^{S297Y}$ OGDH$^{WT}$ OGDH$^{P189L}$ OGDH$^{S297Y}$ OGDH$^{WT}$ OGDH$^{P189L}$ OGDH$^{S297Y}$ Untransfected
anti-FLAG

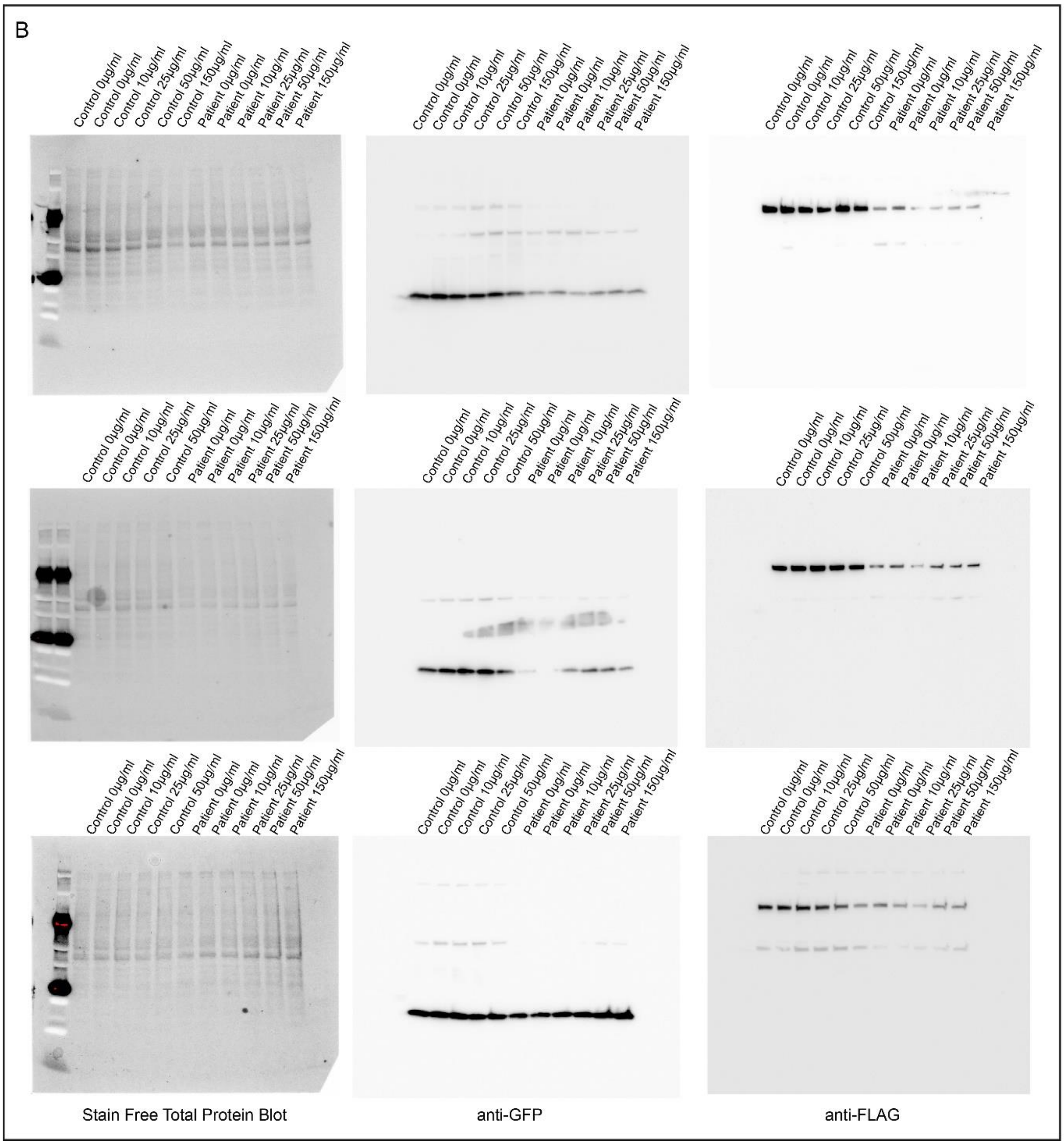


**Supplementary Figure 4 | Full western blot images.** (A) Western blot data corresponding to Figure 2D and Supplementary Figure 3 HEK293 pcDNA3.1 transfections. (B) Western blot data corresponding to Figure 2E and Supplementary Figure 6.

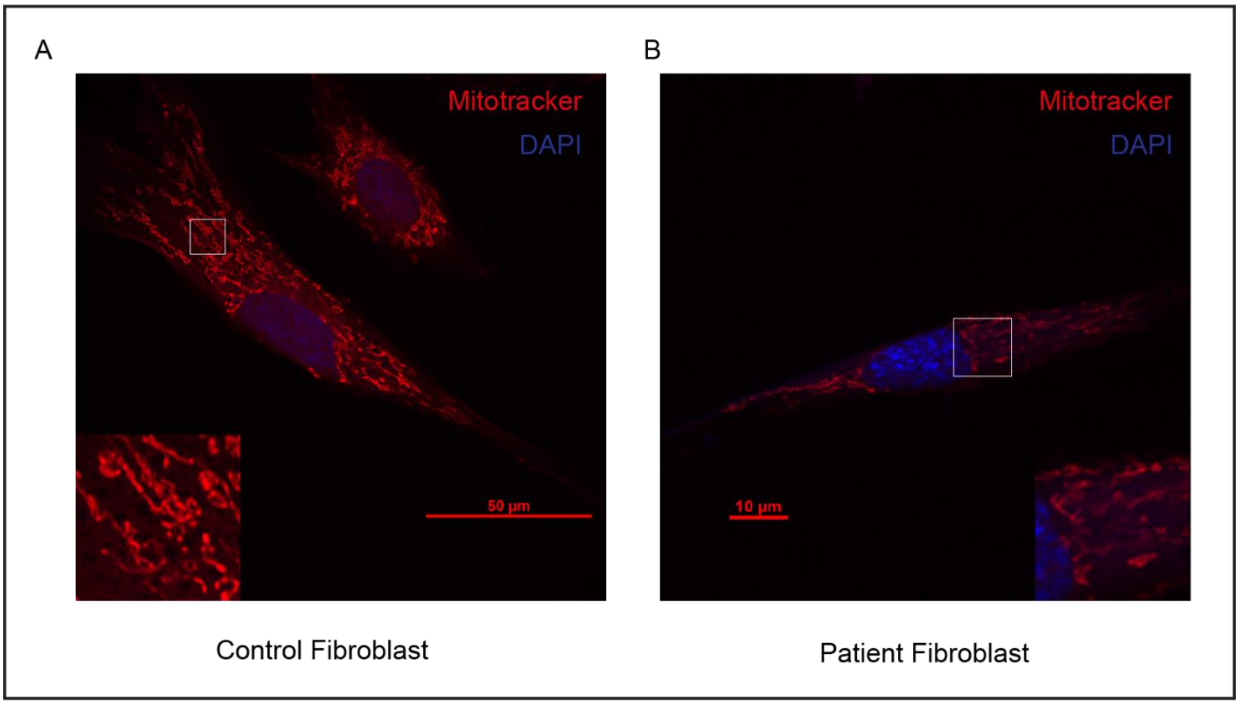


**Supplementary Figure 5 | Visualisation of mitochondria in individual 1 p.(Ser297Tyr) and control fibroblasts using Mitotracker (red) and DAPI (blue) to stain the nucleus.** (A) Healthy control fibroblasts. (B) Individual 1 fibroblasts harbouring the p.(Ser297Tyr) variant.

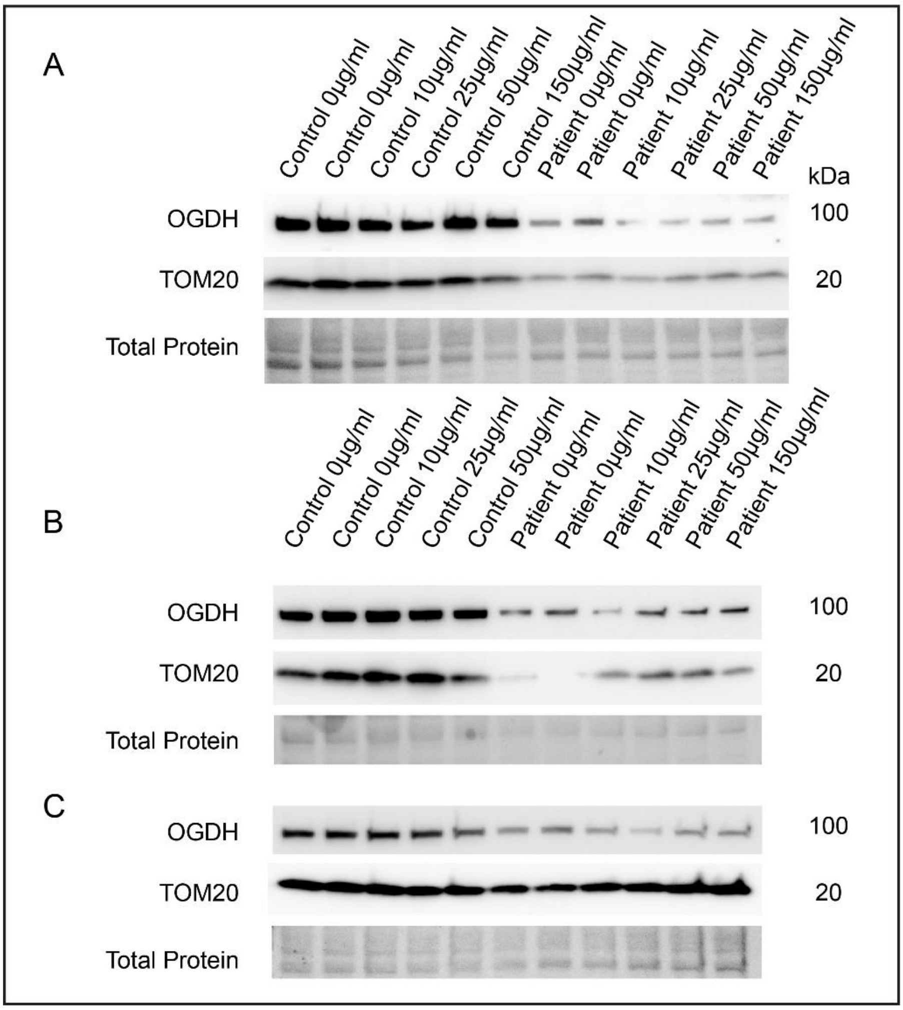


**Supplementary Figure 6 | Replicates of individual 1 p.(Ser297Tyr) and control fibroblast OGDH protein detection following a 24 hour treatment with cycloheximide.** A, B, C showing three separate experiment results. (C) Figure included in the main text body (Figure 2E). 150µg/ml cycloheximide treatment not included in all experiments due to toxicity in control cells.

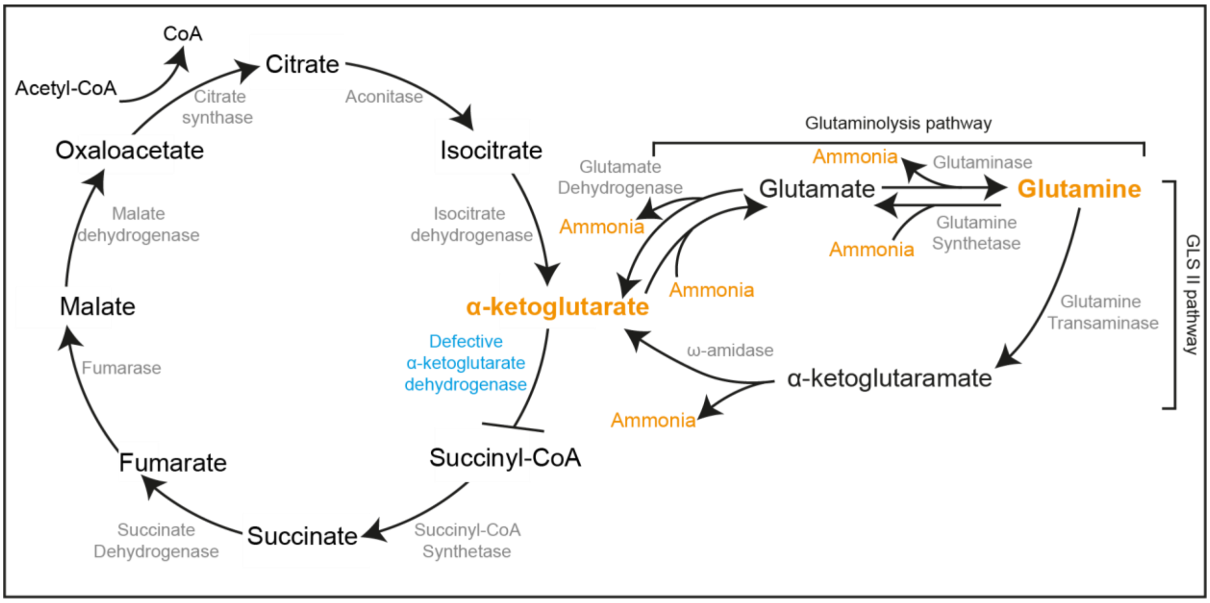


**Supplementary Figure 7 | The TCA cycle and glutamine synthesis pathways.** The TCA cycle with a block at the conversion of α-ketoglutarate to succinyl-coA due to a defective α-ketoglutarate dehydrogenase complex, shown in blue. Next to the TCA cycle, the pathways for the conversion between glutamine and α-ketoglutarate are shown. Metabolites increased in patients are shown in orange.
